# Extracellular Signal-Regulated Kinase Signaling in CD4-Expressing Cells Inhibits Osteochondromas

**DOI:** 10.3389/fimmu.2017.00482

**Published:** 2017-05-01

**Authors:** Marie Wehenkel, Maripat Corr, Clifford S. Guy, Benjamin A. Edwards, Ashley H. Castellaw, Christopher Calabrese, Gilles Pagès, Jacques Pouysségur, Peter Vogel, Maureen A. McGargill

**Affiliations:** ^1^Department of Immunology, St. Jude Children’s Research Hospital, Memphis, TN, USA; ^2^Division of Rheumatology, Allergy, and Immunology, University of California San Diego, La Jolla, CA, USA; ^3^Department of Veterinary Pathology, St. Jude Children’s Research Hospital, Memphis, TN, USA; ^4^Institute for Research of Cancer and Aging (IRCAN), University of Nice Sophia-Antipolis, Nice, France; ^5^Centre Scientifique de Monaco (CSM), Monaco, France

**Keywords:** cartilage, extracellular signal-regulated kinase, osteochondroma, chondrocyte, animal model, bone

## Abstract

Defects in cartilage homeostasis can give rise to various skeletal disorders including osteochondromas. Osteochondromas are benign bone tumors caused by excess accumulation of chondrocytes, the main cell type of cartilage. The extracellular signal-regulated kinase (ERK) pathway is a major signaling node that functions within chondrocytes to regulate their growth and differentiation. However, it is not known whether the ERK pathway in other cell types regulates cartilage homeostasis. We show here that mice with a germline deficiency of *Erk1* and a conditional deletion of *Erk2* in cells that express CD4, or expressed CD4 at one point in development, unexpectedly developed bone deformities. The bone lesions were due to neoplastic outgrowths of chondrocytes and disordered growth plates, similar to tumors observed in the human disease, osteochondromatosis. Chondrocyte accumulation was not due to deletion of *Erk2* in the T cells. Rather, *CD4cre* was expressed in cell types other than T cells, including a small fraction of chondrocytes. Surprisingly, the removal of T cells accelerated osteochondroma formation and enhanced disease severity. These data show for the first time that T cells impact the growth of osteochondromas and describe a novel model to study cartilage homeostasis and osteochondroma formation.

## Introduction

The differentiation and proliferation of cartilage cells is a highly ordered and tightly regulated process that is essential for bone growth. Defects in this process can cause a variety of disorders including osteochondromas, which are the most common type of benign bone tumor. These tumors are caused by excess accumulation of chondrocytes that erupt from the growth plate and are associated with significant pain, restriction of movement, impingement of blood vessels and nerves, and severe deformities. The most severe complication is malignant transformation into a secondary chondrosarcoma. Osteochondromas typically develop during childhood and adolescence as single tumors, although multiple tumors can develop in the heritable disease, multiple hereditary exostoses, also known as osteochondromatosis (1). The molecular and cellular mechanisms that maintain cartilage homeostasis and prevent osteochondroma formation are incompletely understood.

The extracellular signal-regulated kinase (ERK) pathway is transiently activated in response to extracellular cues in almost every type of cell. ERK is downstream of several growth factor receptors and is critical for cell-cycle progression, differentiation, lineage specification, and cell survival (2). Contrary to its role in most cell types, ERK activation decreases chondrocyte proliferation (3–5). In fact, sustained ERK signaling was associated with mutations that led to reduced chondrocyte proliferation and achondroplasia, a form of dwarfism (3, 6). On the other hand, inactivation of ERK signaling in chondrocytes led to an expansion of hypertrophic chondrocytes and enhanced growth of cartilaginous skeletal elements (5). These data suggest that ERK signaling within chondrocytes suppresses proliferation. In addition, deletion of *Erk1* and *Erk2* in osteo-chondroprogenitor cells blocked osteoblast differentiation and caused an accumulation of hypertrophic chondrocytes, indicating that ERK signaling is also important in lineage commitment and differentiation of chondrocytes (7).

While there is an intrinsic role for ERK signaling in the proliferation and differentiation of chondrocytes, it is not known whether this pathway is also important in other cells types to regulate cartilage homeostasis. We generated mice with a germline deletion of *Erk1* and a conditional deletion of *Erk2* mediated by *CD4cre* [*Erk1^−/−^.Erk2^fl/fl^.CD4cre*^+^, double knockout (DKO^CD4^)] (8, 9). *CD4cre* is expressed in all T cell subsets during development in the thymus, and therefore deletes genes in CD4^+^ T cells, CD8^+^ T cells, and NKT cells. Unexpectedly, we found that the DKO^CD4^ mice developed bone deformities due to hyperplastic outgrowths of epiphyseal chondrocytes. The bone lesions lacked mononuclear infiltrate and resembled the human disease, osteochondromatosis. Interestingly, removing the T cells by breeding the DKO^CD4^ mice to *Rag1^−/−^* mice did not prevent osteochondroma formation. In fact, tumor incidence and severity was enhanced in DKO^CD4^ mice lacking T cells, indicating that T cells play a role in regulating chondrocyte accumulation. While T cells are known to contribute to the destruction of cartilage in rheumatoid arthritis and other inflammatory bone disorders, thus far, a role for immune cells in the growth of cartilage or osteochondromas has not been described.

## Materials and Methods

### Mice

*Erk1^−/−^.Erk2^fl/fl^.CD4cre*^+^, double knockout (DKO^CD4^) mice were provided by Stephen Hedrick. 129.DKO^CD4^ mice were backcrossed to the 129 background for 6 generations and B6.DKO^CD4^ mice were backcrossed to the C57BL/6 strain for 12 generations. The ROSA.LSL.TdTomato (tdTom) reporter mice on the C57BL/6 background were provided by Dario Vignali and crossed to the N6 129.DKO^CD4^ mice. The offsprings were then intercrossed. The ROSA.LSL.YFP reporter mice on the C57BL/6 background were obtained from Jackson Laboratories and crossed to the N6 129.DKO^CD4^ mice. N6 129.DKO^CD4^ mice were crossed to C57BL/6 *Rag1^−/−^* mice (Jackson Laboratories), and the offsprings were intercrossed. Littermate controls (LMC) were used for all experiments. All mice were housed under specific pathogen-free conditions at St. Jude Children’s Research Hospital. Animal studies met the approval of the Animal Ethics Committee.

### Incidence and Disease Severity Scoring

Mice of the indicated genotypes were monitored for disease incidence on a weekly basis. The symptoms tracked included swollen or deformed bones, abnormal gait, hunched posture, and dyspnea. Two consecutive weeks of a symptom being displayed, continuing through the remainder of the experimental observation period, was required to indicate disease incidence. Incidence was then denoted as the age at the first symptomatic observation. Disease severity was determined at the humane or experimental endpoint by addition of the number of symptomatic features, which could be detected visually, along with an additional value given for severe symptoms.

### *In Vivo* CT

Mice were imaged using a 2,048 mm × 3,072 mm field of view (FOV) with whole body coverage achieved using automatic stitching of two bed positions (20% overlap). Projections were acquired at 80 kVp and 500 µA (165 ms exposure) over a rotation of 195° (390 steps) providing an isotropic resolution of 107 µm. Data were processed using Inveon Research Workplace (IRW) software for visualization. Isosurface (ISO) rendering is a raycast method where surfaces of similar density objects are rendered and the remaining materials are hidden. Maximum intensity projection (MIP) is a method where the color (in this case, white balance of grayscale) of the densest object along each raycast path is used to determine the final color of each pixel.

### *Ex Vivo* CT

μCT micrographs of selected bones were generated using a specimen μCT scanner (LocusSP Specimen CT, GE Healthcare) at 14 µm isotropic voxel size, with 500 projections, 4 averages, an integration time of 3,000 ms, photon energy of 80 keV, and a current of 80 µA. Data were processed using MicroView software for visualization and bone morphometry quantification. Bone lengths were determined using manual electronic calipers built into the MicroView software system. For bone morphometry analysis, volumes of interest (VOIs) were generated covering 2.1 mm (proximal and distal femur and distal humerus) or 1.4 mm (proximal humerus). In-plane cortical bone thickness was manually determined at two independent points within the slice and three slices representative of proximal, medial, and distal components of the VOI. For cortical bone processing, images were automatically thresholded to select for cortical bone, segmented to this threshold, and cortical bone thickness across the VOI calculated. For bone volume and trabecular analyses, cortical bone was blanked from the VOI and whole volumes evaluated.

### Histology

Bones were fixed in 10% neutral buffered formalin overnight and then decalcified in TBD-2 (Thermo Fisher Scientific, Waltham, MA, USA) for three days. The tissues were processed routinely, embedded in paraffin, sectioned at 4 µm, and stained with hematoxylin and eosin, Safranin O, or Alcian blue. Bone sections were examined by an experienced veterinary pathologist.

### Pathology

Full necropsies were performed on selected animals [B6.LMC (*n* = 11), B6.DKO^CD4^ (*n* = 3); 129.LMC (*n* = 8), 129.DKO^CD4^ (*n* = 6)]. In addition to bones, the following tissues were examined: heart, skeletal muscle, tongue, trachea, lung, thyroid, liver, kidney, adrenal gland, salivary gland, lymph node, white adipose, brown adipose, aorta, thymus, spleen, pancreas, stomach, duodenum, jejunum, ileum, cecum, colon, urinary bladder, skin, brain, eye, nose, teeth, ear, bone, bone marrow, and either testis, epididymis, prostate gland, seminal vesicle, and vas deferens, or uterus and ovary.

### *In Vivo* Radiography

Animals were anesthetized using avertin, and data were acquired at 50 µm^2^ in-plane resolution with 26 kV energy and ~12 s exposure time. Prone position data were acquired at shelf position 2 [beam coverage diameter (BC) 27.7 cm, source to object distance (SOD) (28.6 cm)], and lateral position data were acquired at shelf position 4 (BC = 10.4 cm, SOD = 14.3).

### Chondrocyte Isolation and Analysis

Five- to seven-day-old mice were harvested individually for isolation and culture of chondrocytes as previously described (10). In addition, osteochondromas were identified in mice greater than 20 weeks of age by planar X-ray and microdissected. PCR was performed as previously described (8). Cells were lysed for western blotting using NP-40 lysis buffer with protease and phosphatase inhibitor cocktails (Calbiochem), and protein concentration was determined by BCA assay (Thermo Scientific). Equal protein concentrations (40 µg) were then denatured and separated by 10% polyacrylamide SDS gel electrophoresis (BioRad). After transfer to PVDF membrane, antibodies to ERK1/2 (Zymed 61-7400), COL2A1 (Santa Cruz M2139), and β-actin (Sigma AC-74) were used for analysis.

### Immunofluorescence

Mice were euthanized *via* CO_2_ asphyxiation and perfused with 4% paraformaldehyde (Electron Microscopy Sciences) and the hindlimbs were bulk demuscled. Hindlimbs (femurs, tibiae, and fibulae) were fixed in 4% paraformaldehyde overnight at 4°C prior to decalcification with 0.5 M EDTA for an additional 24 h at 4°C. Samples were cryoprotected with 20% sucrose in PBS prior to embedding in tissue freezing medium (Electron Microscopy Sciences). Twenty micrometer cryosections were air dried for 1 h at room temperature prior to incubation with AF488-labeled wheat germ agglutinin (WGA) (Life Technologies; 2 µg/ml) for 1 h at room temperature. Slides were washed in TBS prior to mounting with hardset media containing DAPI (Vector Laboratories).

Widefield fluorescent images were acquired using a Nikon TiE microscope equipped with a Lambda LS lightsource (Sutter Instruments), 10 × 0.45 NA or 20 × 0.75 NA Plan Apo objectives, DU-897 EMCCD camera (Andor Technology), and standard DAPI/FITC/TRITC/Cy5 filter sets. Image analysis was performed using NiS Elements AR software (Nikon).

Confocal microscopy was performed using an AxioObserverZ.1 inverted microscope (Zeiss) equipped with a CSU-22 spinning disk (Yokagawa), 405, 473, 561, and 660 nm laser lines, and Evolve EMCCD camera (Photometrics). Images were acquired and analyzed using Slidebook software (Intelligent Imaging Innovations).

### Flow Cytometry

Tissues were harvested and processed as described previously (11). After incubation with anti-FcReceptor, cells were stained with CD44-FITC, CD8α-PerCP (eBioscience), CD69-APC-Cy7, CD19-BV510, and CD4-BV711 (BioLegend) for 20 min on ice. For intracellular staining of FoxP3-PE (eBioscience), cells were fixed and permeabilized with Mouse Foxp3 Buffer set (BD Biosciences) after surface staining, according to the manufacturer’s instructions. Samples were run on a BD LSRFortessa analyzer and the results evaluated in FlowJo. Data were plotted in GraphPad Prism for statistical analysis.

## Results

### DKO^CD4^ Mice Develop Deformed Bones in Multiple Locations

**Figure 1:**
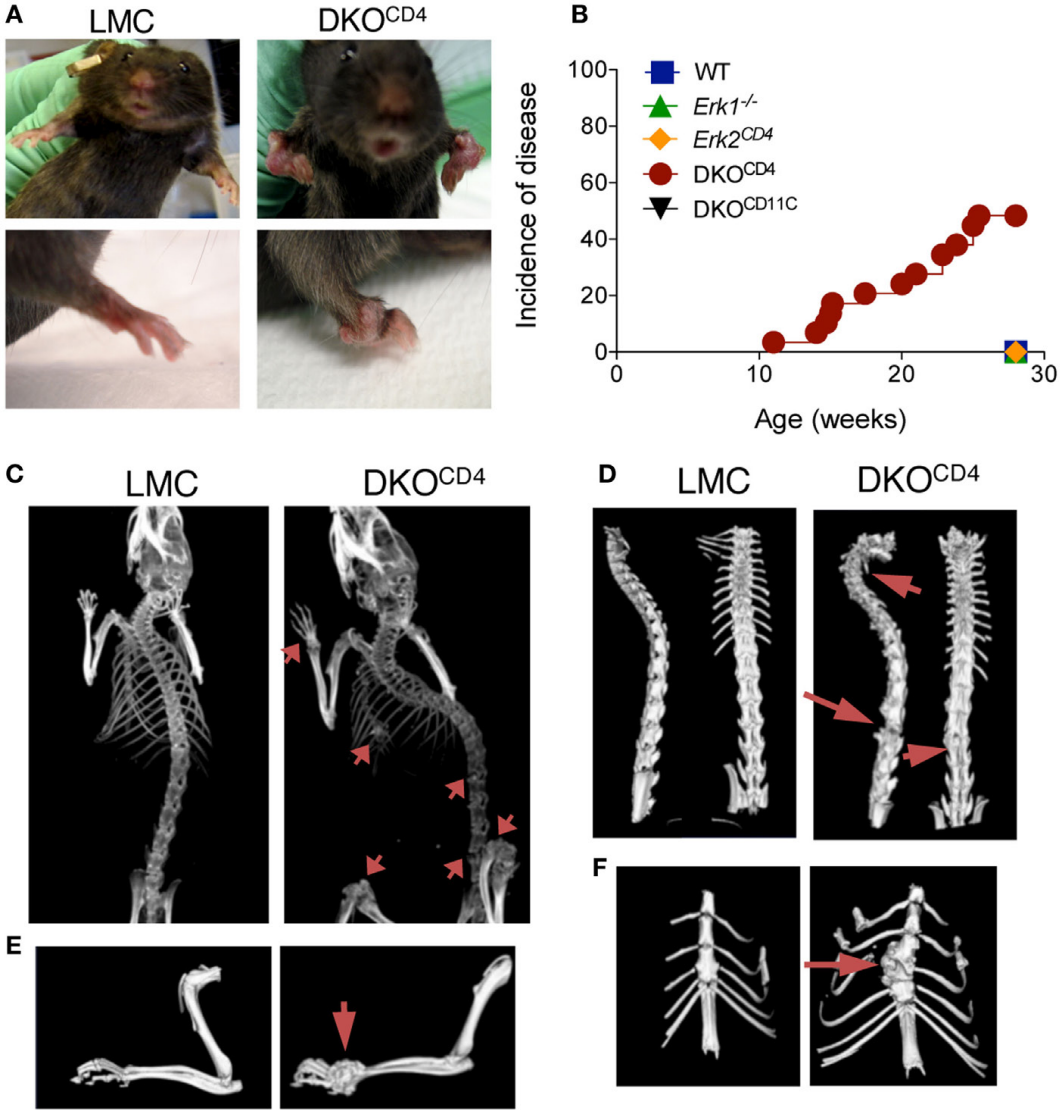
**Joint deformities develop in DKO^CD4^ mice**. **(A)** Anatomical deformities in the joints of a DKO^CD4^ mouse and littermate control (LMC) are pictured. **(B)** Mice were monitored weekly for swollen or deformed bones, abnormal gait, hunched posture, and dyspnea. The incidence of one of these symptoms is plotted for 29 DKO^CD4^ mice and at least 11 mice in the other groups. Data were analyzed with a Log-rank (Mantel–Cox) test. *P < 0.05 for DKO^CD4^ mice versus all other groups. **(C)** X-ray computed tomography (CT) *in vivo* images of LMC and DKO^CD4^ mice. Arrows indicate lesions in the radius, ulna, tibia, femur, vertebra, and sternum. **(D)** Isosurface rendering of *ex vivo* CT images of the vertebra, **(E)** forelimb, and **(F)** sternum. These images are representative of at least six mice per group.

Mice with a germline deletion of *Erk1* were bred to mice with a conditional deletion of *Erk2* mediated by *CD4cre* [*Erk1^−/−^.Erk2^fl/fl^.CD4cre*^+^, double knockout (DKO^CD4^)] (8, 9). *CD4cre* is expressed in developing T cells, and therefore deletes genes in all T cell subsets. Due to the requirement of ERK signaling in T cell development, DKO^CD4^ mice have a significant reduction in the number of T cells in the spleen and lymph nodes (Figures S1A,B in Supplementary Material) (8, 9). The residual T cells in the DKO^CD4^ mice exhibited an activated phenotype, likely due to the lymphopenic environment and homeostatic proliferation. While there was a reduction in the total number of regulatory T cells, a higher proportion of the CD4^+^ T cells expressed Foxp3 in the DKO^CD4^ mice compared to wild-type mice (Figure S1C in Supplementary Material), which is consistent with the fact that ERK2 signaling suppresses Foxp3 expression (12). Surprisingly, the DKO^CD4^ mice developed noticeable bone deformities between 10 and 20 weeks of age (Figure 1A). Approximately 40% of the DKO^CD4^ mice displayed grossly visible signs of disease by 28 weeks of age (Figure 1B). Importantly, neither *Erk1^−/−^* nor *Erk2^CD4^* single knockout mice developed any signs of disease. Furthermore, *Erk1^−/−^* mice bred to mice with a conditional deletion of *Erk2* mediated by *CD11c-cre* (DKO^CD11c^) did not develop disease (Figure 1B). As there was no bone phenotype in single knockout or wild-type mice, we used these mice interchangeably as littermate controls (LMC) in the remaining experiments.

X-ray computed tomography (CT) revealed pronounced areas of abnormal bone growth in various locations (Figure 1C), including the spinal column (Figure 1D), the radius and ulna (Figure 1E), and the sternum (Figure 1F). Virtual slicing through these deformities demonstrated that the lesions were contiguous with the bone and thus, likely arose from within it (Figure S2 in Supplementary Material).

### Bone Lesions Are Composed of Chondrocytes

**Figure 2:**
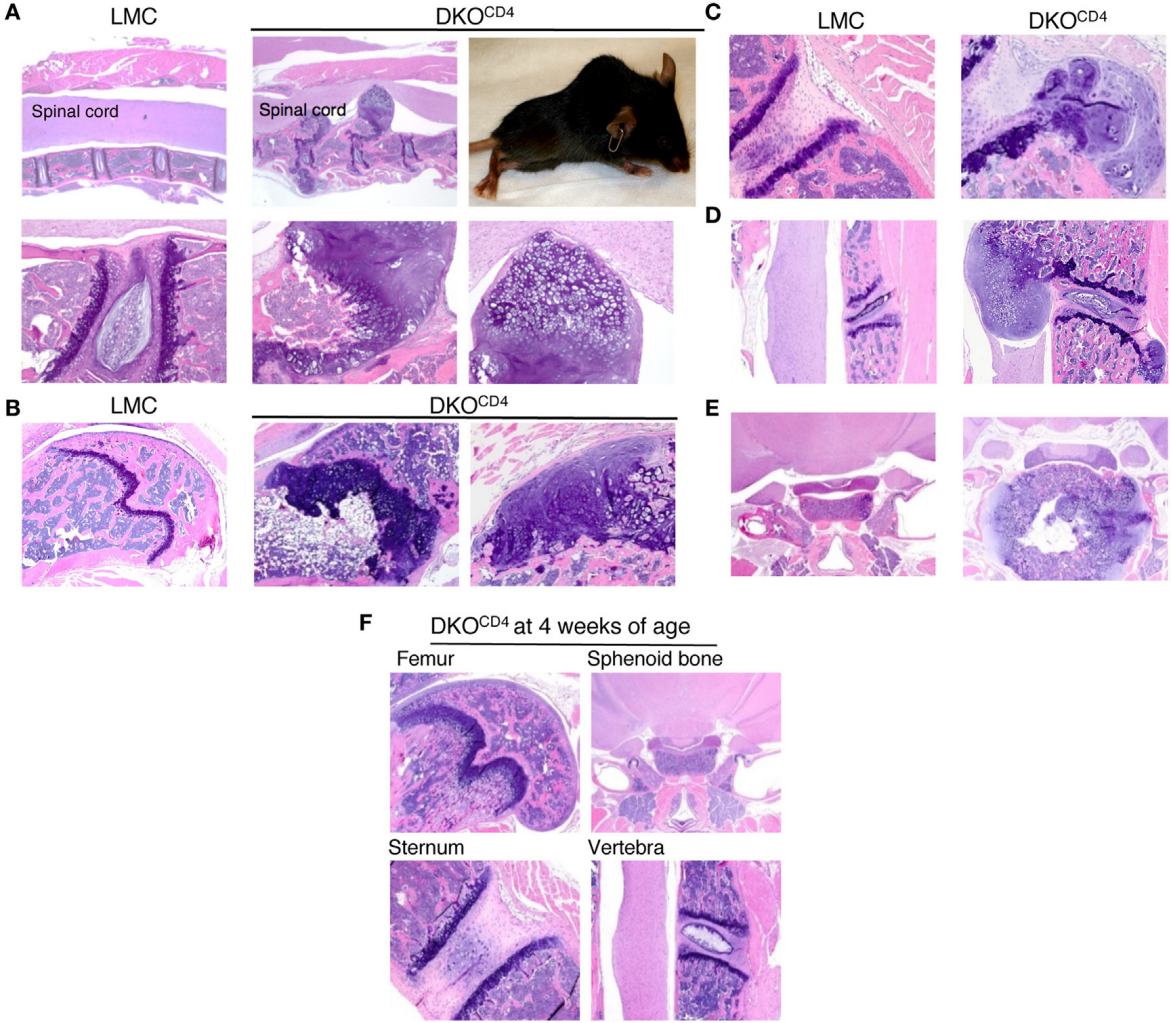
**DKO^CD4^ mice exhibit ectopic accumulation of chondrocytes**. Bones were removed from LMC and 129.DKO^CD4^ mice, fixed, decalcified, and stained with hematoxylin and eosin (H&E). **(A)** Sections of spinal column [2× (top) and 10× (bottom)], including a photograph of the paralyzed DKO^CD4^ mouse from which they were obtained, are shown. Other sections displayed include the **(B)** femur [4× and 10× (far right)], **(C)** sternum (10×), **(D)** vertebra (4×), and **(E)** sphenoid bone (2×). **(F)** H&E analysis of bones is depicted from 4-week-old 129.DKO^CD4^ mice, showing femur (4×), sphenoid bone (2×), sternum (10×), and vertebra (4×). Images are representative of at least five mice.

Histological examination of the DKO^CD4^ mice revealed nodular, disorganized accumulation of cartilage protruding from the bones (Figures 2A–E). The cartilage protrusions were continuous with the epiphyseal growth plate, and the intact perichondrium was visible in multiple lesions, suggesting that the cells that make up these protrusions are epiphyseal chondrocytes. Safranin O and Alcian blue staining confirmed that these lesions were chondrocytic, and showed the absence of non-cartilaginous cells within the lesion (Figure S3 in Supplementary Material). The presence of lesions in the radius, ulna, and sphenoid bone indicates these protrusions did not originate from the annulus fibrosis, which is absent from these locations. Notably, these nodules lacked mononuclear infiltrates, which was surprising as *Erk2* deletion is mediated by *CD4cre*, which is primarily expressed in T cells. Overall, this pathology resembles the human disease, osteochondromatosis.

Histological analysis of a cohort of DKO^CD4^ mice showed that 70% of the DKO^CD4^ mice had at least one osteochondroma, which was significantly higher than the 40% incidence determined by physical examination (Figure 1B; Table 1). As in humans, the bones affected by the tumors varied between DKO^CD4^ mice and had the potential to cause severe morbidity. For example, lesions in the vertebrae caused paralysis by compressing the spinal cord (Figure 2A).

**Table 1:** **Histopathological incidence of bone lesions at 23–44 weeks of age**.

Location of osteochondromas	B6.LMC	B6.DKO^CD4^	129.LMC	129.DKO^CD4^
Any location	0/16	7/10	0/10	12/14
Hindlimb	0/16	7/10	0/10	9/14
Spinal column	0/16	5/10	0/10	11/14
Forelimb	0/16	3/10	0/9	3/13
Carpus	0/16	2/10	0/9	4/13
Head	0/9	1/5	0/5	5/5
Pelvic/femoral joint	0/16	1/10	0/9	0/13
Sternum	0/16	1/10	0/10	7/14
Tail	0/16	1/10	0/10	2/14
Forepaw	0/16	0/10	0/8	0/12
Tarsus	0/16	0/10	0/9	0/13
Hindfoot	0/16	0/10	0/9	0/13

There were no obvious abnormalities in the growth plates or chondrocytes of 4-week-old DKO^CD4^ mice (Figure 2F), indicating that excessive chondrocyte accumulation does not occur early in DKO^CD4^ mice, unlike other models of osteochondromatosis where ERK signaling is disrupted within the chondrocytes (5, 7, 13). Together, these data indicate that germline deletion of *Erk1* along with *CD4cre*-mediated deletion of *Erk2* causes excessive accumulation of chondrocytes, resulting in osteochondromas and disorganized growth plates.

### DKO^CD4^ Mice Do Not Display Overt Signs of Inflammation

**Figure 3:**
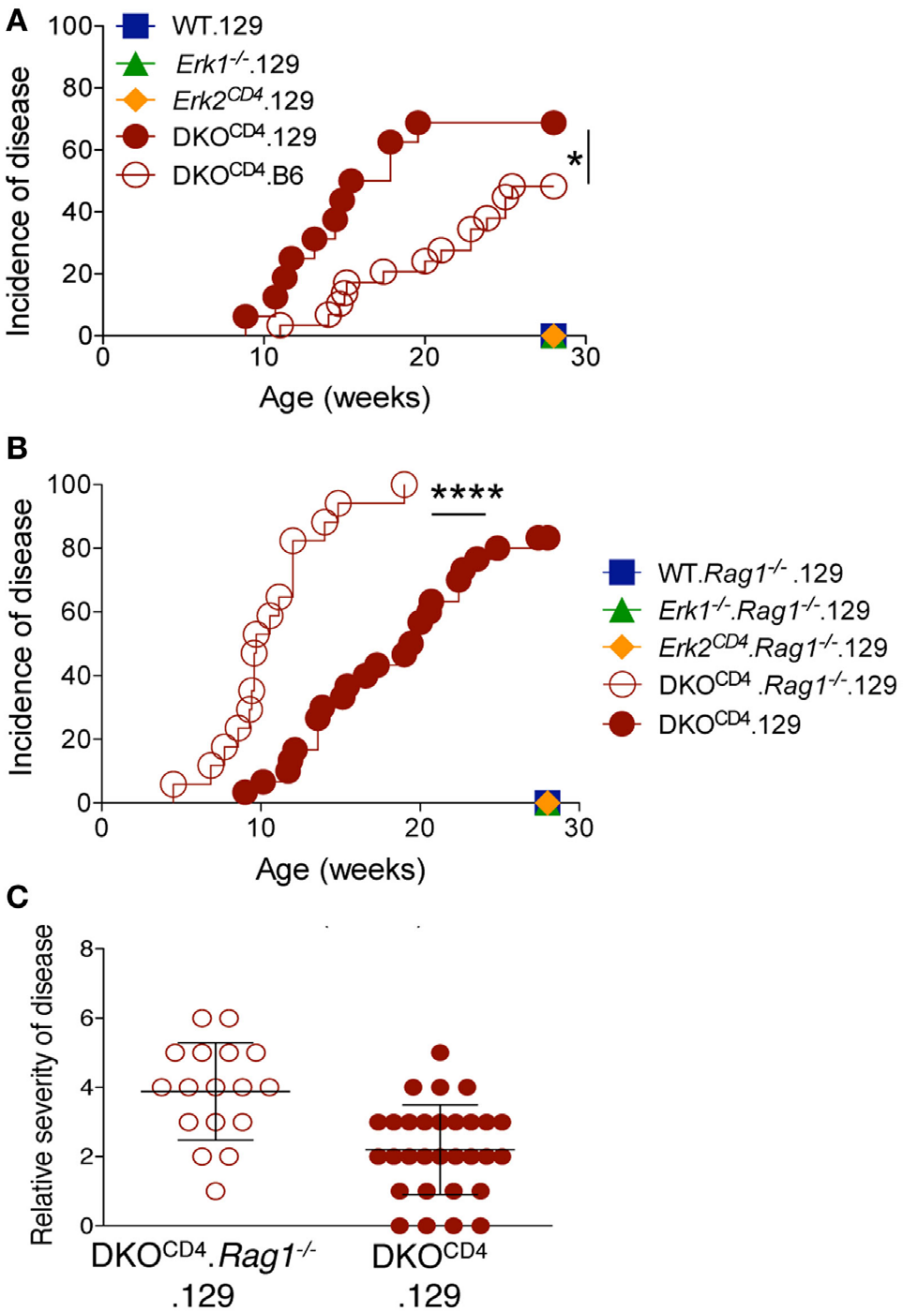
**Osteochondroma formation is not due to deletion of *Erk2* in T cells**. **(A)** DKO^CD4^ mice were crossed to the 129 background and monitored as in Figure 1B. The incidence of symptoms is plotted for 16 129.DKO^CD4^ mice, 29 B6.DKO^CD4^ mice, and at least seven mice in the other groups. Data were analyzed with a Log-rank (Mantel–Cox) test (*P < 0.05). **(B)** 129.DKO^CD4^ mice were bred to *Rag1^−/−^* mice to create DKO^CD4^.*Rag1^−/−^* mice, which were monitored for disease. The incidence of symptoms is plotted for 30 129.DKO^CD4^ and 17 DKO^CD4^.*Rag1^−/−^* mice. Data were analyzed with a Log-rank (Mantel–Cox) test (*P < 0.0001). **(C)** Relative disease severity scores of the mice depicted in Figure 3B.

Crossing the DKO^CD4^ mice onto the 129 background for two generations significantly exacerbated disease, resulting in 73% incidence by 28 weeks of age as determined by manual examination (Figure 3A), and 86% when analyzed by histology (Table 1). Since 129 mice typically exhibit more inflammation in various disease models compared to B6 mice (14–16), we examined all tissues from the B6.DKO^CD4^ and 129.DKO^CD4^ mice for evidence of mononuclear infiltrate or inflammation. Despite a thorough analysis, there was no indication of overt inflammation on either background. In addition, there was not an increase in anti-nuclear (ANA) antibodies, double-stranded DNA (dsDNA) autoantibodies, or inflammatory cytokines in the serum from 129.DKO^CD4^ mice compared to LMC (data not shown). These data indicate that there was no apparent inflammation in the 129.DKO^CD4^ mice that is typically associated with T cell-mediated autoimmunity.

### Osteochondroma Formation Is Not Due to Deletion of *Erk2* in T Cells

Since the majority of cells that express CD4 are T cells, we tested whether deletion of *Erk2* in the T cells induced osteochondromas by removing the T cells in DKO^CD4^ mice. DKO^CD4^ mice were bred to *Rag1^−/−^* mice to create DKO^CD4^.*Rag1^−/−^* mice, which lack T cells. Surprisingly, the DKO^CD4^.*Rag1^−/−^* mice not only developed osteochondromas, but these mice developed tumors faster, and with increased severity compared to DKO^CD4^ mice (Figures 3B,C). These data indicate that the osteochondromas are not due to deletion of *Erk2* in T cells, and additionally that T cells may suppress the formation of osteochondromas. Furthermore, deletion of *Erk2* in a cell type other than T cells induces osteochondroma formation. Subsets of myeloid cells and innate lymphoid cells also express CD4, and it is possible that deletion of *Erk2* in these cells may result in dysregulation of growth factors, which causes excessive chondrocyte accumulation.

### Majority of Chondrocytes Express ERK2 in DKO^CD4^ Mice

**Figure 4:**
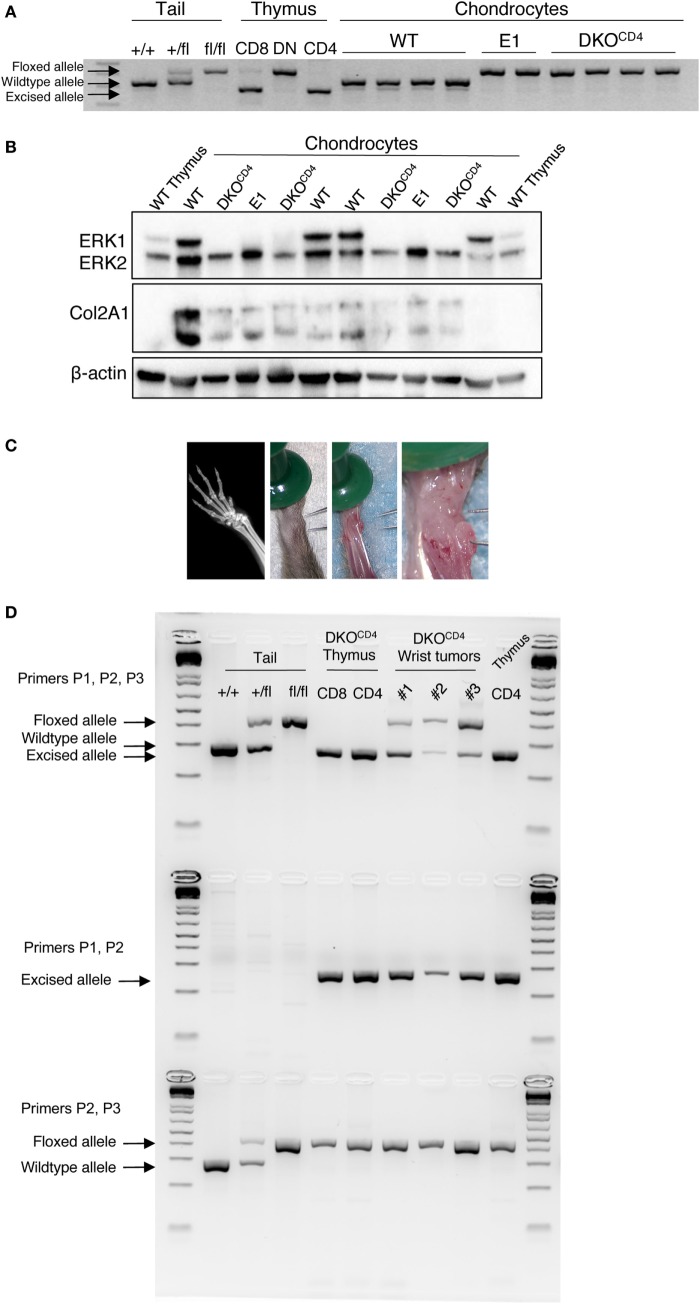
**ERK2 is expressed in the majority of chondrocytes in DKO^CD4^ mice**. **(A)** Chondrocytes were isolated from individual WT, *Erk1^−/−^.Erk2^fl/fl^.CD4cre^−^* (E1), or DKO^CD4^ mice and cultured *in vitro*. DNA was isolated and analyzed by PCR for the presence of the wild-type, floxed, or excised *Erk2* allele. Tail DNA is shown as controls for the wild-type and floxed alleles, while DNA from thymocytes serves as the positive control for the excised allele. **(B)** Cultured chondrocytes were lysed and analyzed by western blot for ERK1, ERK2, type II collagen (Col2A1), and beta-actin. **(C)** Planar X-ray (left) and surgical microscopy images of a representative lesion from a DKO^CD4^ mouse. **(D)** Three microdissected tumors from individual mice greater than 20 weeks of age were analyzed by PCR to determine whether the *Erk2* gene was wild-type, floxed, or excised by Cre recombinase. DNA from DKO^CD4^ thymocytes is shown as a positive control for *Erk2* excision, while tail DNA is a negative control for excision. The first row contains primers that amplify the wild-type, floxed, or excised alleles, the second row is primers that only amplify the excised allele. The third row contains primers that amplify the wild-type or floxed alleles.

Contrary to other cell types, ERK activation in chondrocytes is associated with decreased proliferation and differentiation (3, 17). Although chondrocytes in DKO^CD4^ mice are *Erk1*-deficient, ERK2 expression in these cells is sufficient to regulate chondrocyte development, as indicated by the absence of osteochondromas in *Erk1^−/−^* mice. Likewise, other groups found that mice with single deletions of *Erk1* or *Erk2* specifically in chondrocytes did not display skeletal defects (5, 7). While chondrocytes are not known to express the CD4 protein, it was possible that *CD4cre* deleted *Erk2* in chondrocytes, rendering these cells deficient in both *Erk1* and *Erk2*. Therefore, we investigated *Erk2* expression in chondrocytes isolated from wild-type, *Erk1^−/−^.Erk2^fl/fl^.CD4cre^−^* (E1), and DKO^CD4^ mice. Analysis of chondrocyte DNA revealed that the *Erk2* gene was floxed in E1 and DKO^CD4^ chondrocytes; however, it was not excised by Cre recombinase as it was in CD8^+^ and CD4^+^ thymocytes from DKO^CD4^ mice (Figure 4A). Additionally, western blot analysis demonstrated that the ERK2 protein was expressed at similar levels in all chondrocytes examined (Figure 4B).

Further evidence that *Erk2* is not deleted in the majority of chondrocytes in DKO^CD4^ mice is provided by the lack of characteristic bone deformities or alterations in bone length typical of mice that have reduced ERK signaling specifically in the chondrocytes (3, 5, 7). Others demonstrated that mice deficient in *Erk1* and *Erk2* specifically in the chondrocytes died at birth with severe bone deformities (7). In addition, deletion of *Erk1* and *Erk2* in mesenchymal cells (*PrxCre*) of the head and limb caused ectopic cartilage development and severe limb deformity at birth (7). Thus, if *CD4cre* deleted *Erk2* in chondrocytes or osteo-chondroprogenitors, we would expect to see elongated bones, severely deformed bones, or disorganized growth plates in DKO^CD4^ mice beginning at a very young age. However, DKO^CD4^ mice did not have any differences in bone length, trabecular density or thickness, average cortical bone thickness, or the cortical area fraction compared to LMC (Figure S4 in Supplementary Material). Moreover, the growth plates of DKO^CD4^ mice at 4 weeks of age were indistinguishable from LMC (Figure 2F).

### *CD4cre* Deletes Genes in Cells Other Than T Cells

To test whether osteochondromas were due to clonal expansion of rare chondrocytes that deleted the *Erk2* allele, we analyzed chondrocytes from microdissected tumors by PCR that distinguishes the *Erk2* wild-type, floxed, and excised alleles (Figure 4C). These tumors were dissected from mice greater than 20 weeks of age. We found that both the floxed and excised *Erk2* alleles were detected in DNA from the chondrocytes in the tumors, suggesting that some of the chondrocytes deleted *Erk2* (Figure 4D). Deletion of *Ext1* or *ptpn11* (SHP2) in only a proportion of chondrocytes can cause osteochondromas, enchondromas, or exostoses to form (18, 13, 19), indicating that deletion of genes in only a fraction of chondrocytes can cause abnormal accumulation. Thus, it is possible that the presence of a few chondrocytes that delete *Erk2* is sufficient to cause abnormal chondrocyte accumulation and osteochondromas.

**Figure 5:**
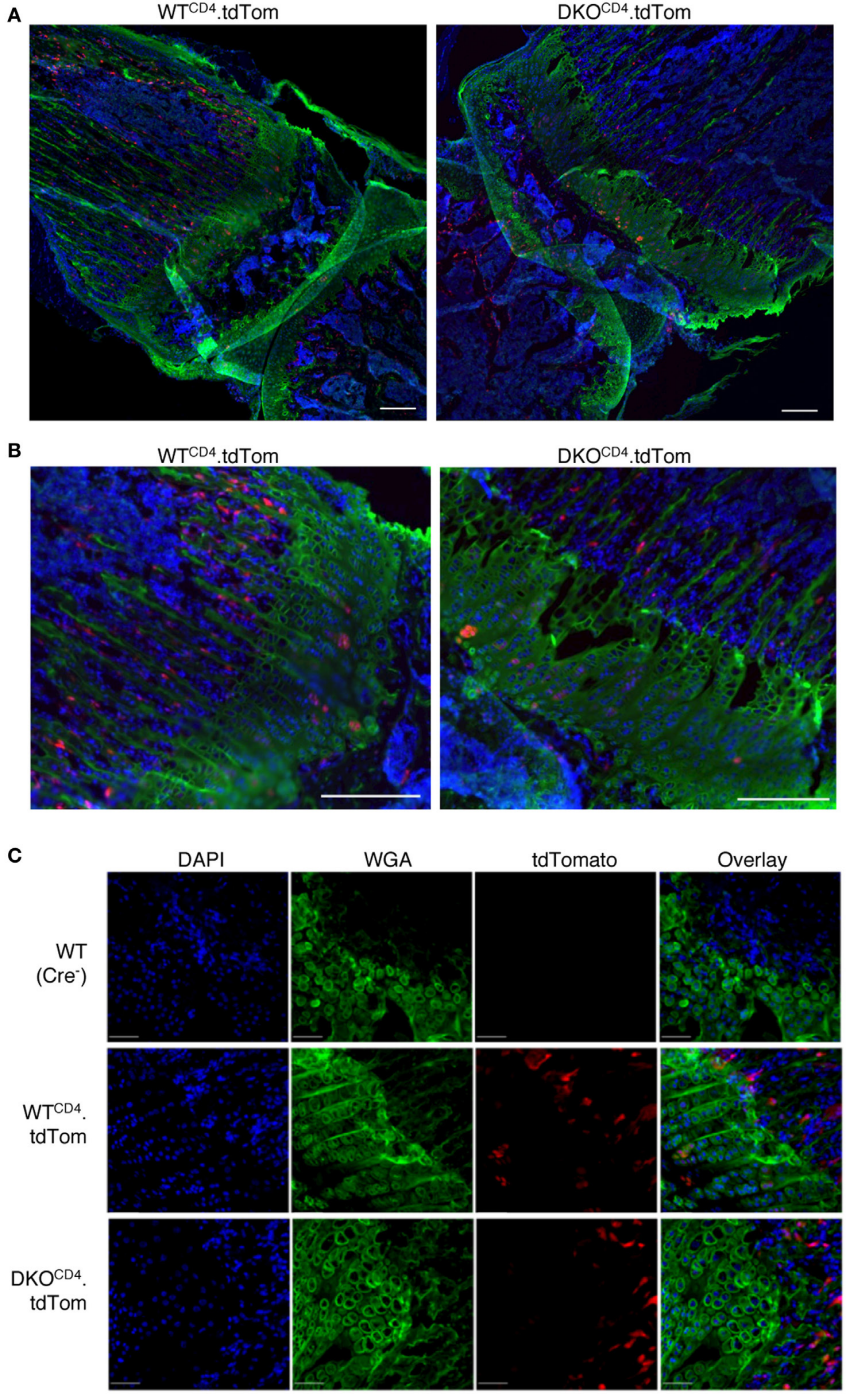
**CD4-lineage positive cells are in close proximity to the epiphyseal growth plate**. Hindlimbs from 5-week-old WT^CD4^.tdTom and DKO^CD4^.tdTom mice were cryosectioned then stained with DAPI and wheat germ agglutinin. **(A)** Composite widefield sections of tibia obtained at 10× were digitally stitched together. **(B)** Sections from **(A)** were electronically zoomed. **(C)** Confocal images of femur sections taken at 20×. Representative images of at least three mice per group are presented.

While we detected the excised *Erk2* allele in DNA from osteochondromas, the PCR does not indicate what proportion of chondrocytes deleted the *Erk2* allele. Therefore, we used Cre reporter mice to investigate what proportion of chondrocytes deleted *Erk2*. DKO^CD4^ mice were bred to ROSA.LSL.tdTomato (tdTom) or ROSA.LSL.YFP reporter mice that express a *loxP*-flanked stop cassette followed by the fluorescent protein. Cells that express *CD4cre* at any point in development excise the stop cassette and express tdTomato or YFP for the lifespan of that cell. Western blot analysis demonstrated that the tdTomato^+^ or YFP^+^ reporter splenocytes lacked ERK2 protein, while the tdTomato-negative or YFP-negative cells from these mice retained expression of ERK2 protein, indicating that the fluorescent reporters are effective indicators of *Erk2* deletion (Figure S5 in Supplementary Material). Sections of hindlimbs of 5-week-old *Erk1*^+^*^/^*^+^*Erk2*^+^*^/^*^+^*.tdTom^fl/fl^.CD4cre*^+^ (WT^CD4^.tdTom) and *Erk1^−/−^Erk2^fl/fl^.tdTom^fl/fl^.CD4cre*^+^ (DKO^CD4^.tdTom) mice were analyzed *via* microscopy by staining with WGA to visualize chondrocytes, and DAPI to identify nuclei. Composite widefield sections showed tdTomato^+^ cells in the bones of both WT^CD4^.tdTom and DKO^CD4^.tdTom mice (Figures 5A,B). While the majority of the chondrocytes did not express the tdTomato reporter, there were tdTomato^+^ cells in the bone marrow and in the area of the chondrocytes in the growth plate. Confocal microscopy also showed that the majority of chondrocytes in the growth plates were not tdTomato^+^; however, a small proportion did express the reporter (Figure 5C). These data suggest that a minor fraction of chondrocytes may be *Erk2-*deficient, which may contribute to osteochondroma formation.

**Figure 6:**
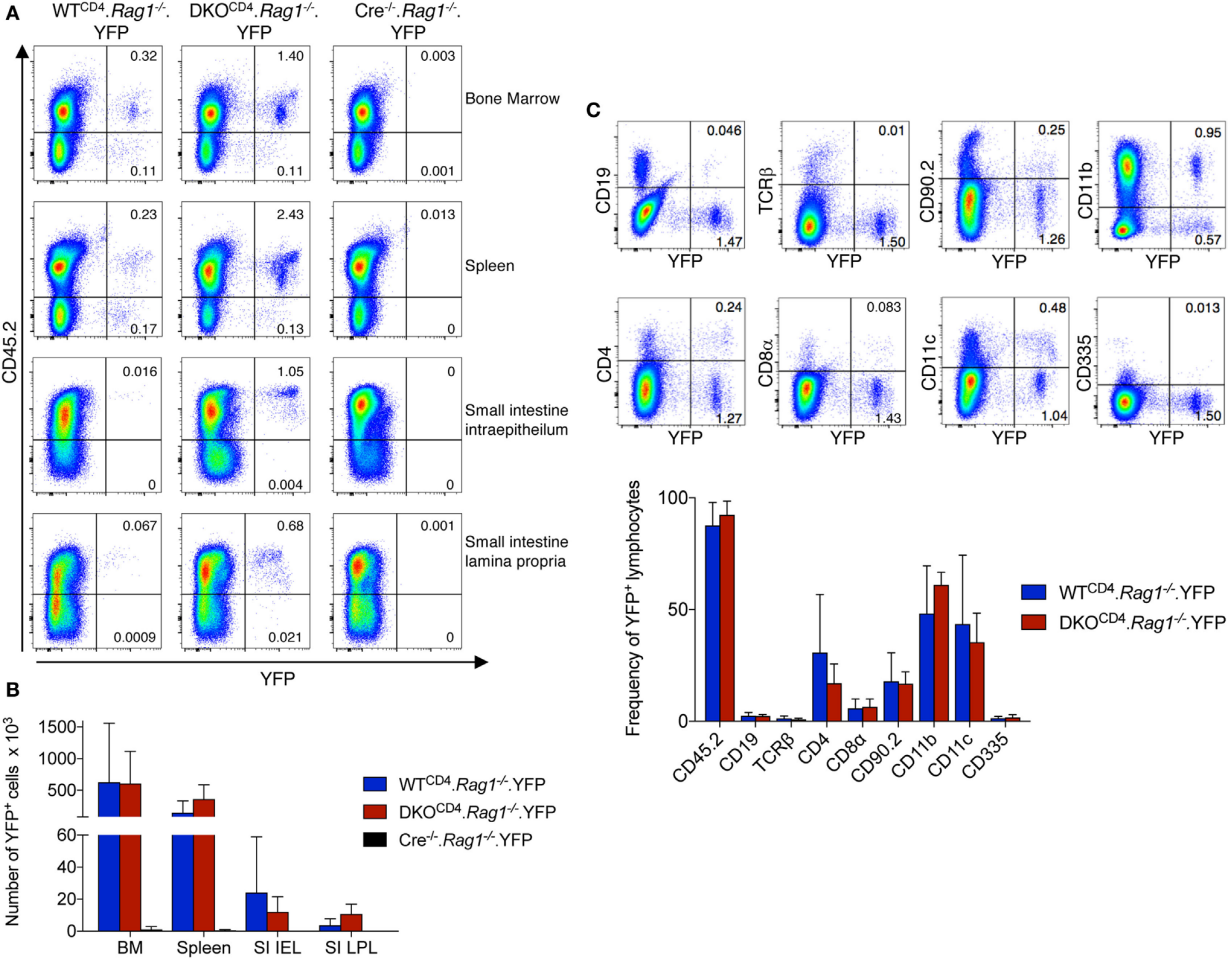
***CD4cre* is expressed in multiple immune cell types**. **(A)** Lymphocytes from the bone marrow, spleen, small intestine intraepithelium, and small intestine lamina propria of WT^CD4^.*Rag1^−/−^* and DKO^CD4^.*Rag1^−/−^* mice were examined for expression of YFP by flow cytometry. Representative dot plots are shown with frequencies of YFP^+^ populations of total lymphocytes based on forward and side scatter. Cre^−/−^.*Rag1*^−/−^ mice are shown as a negative control for YFP expression. **(B)** Quantification of the total numbers of YFP^+^ cells per tissue from plots shown in **(A)**. *n* = 4 mice per group for WT^CD4^.*Rag1^−/−^* and DKO^CD4^.*Rag1^−/−^* mice and *n* = 2 for the Cre*^−^*^/^*^−^*.*Rag1^−/−^* group. **(C)** Representative dot plots from the bone marrow of a DKO^CD4^.*Rag1^−/−^* mouse are shown. Cells are electronically gated on lymphocytes based on forward and side scatter, and single cells. Numbers represent frequencies of YFP^+^ populations of the lymphocyte population. The average proportion of YFP^+^ cells that is positive for each subset in the bone marrow is plotted in the graph (*n* = 4 per group).

To further assess what cells other than T cells express *CD4cre*, we bred DKO^CD4^.*Rag1^−/−^* mice to the ROSA.LSL.YFP reporter mouse to create *Erk1^−/−^Erk2^fl/fl^.Rag1^−/−^*.*YFP^fl/fl^.CD4cre*^+^ (DKO^CD4^.*Rag1^−/−^*.YFP) mice and analyzed cells from the bone marrow, spleen, and small intestine intraepithelial (IEL) and lamina propria (LP) for YFP expression. Although the proportion of YFP^+^ cells was low in all of the organs, an YFP^+^ population was consistently present in all of the mice we analyzed (Figure 6A). The degree of *CD4cre* expression varied between mice, but was not influenced by the absence of *Erk2* (Figure 6B). The majority of YFP^+^ cells in the bone marrow were CD45^+^ and either CD11b^+^ or CD11c^+^ (Figure 6C). The YFP^+^ cells were not Thy1^+^, suggesting that they were not ILCs. Additionally, DKO^CD4^.YFP mice that were *Rag1*-sufficient also had a significant proportion of cells other than T cells that expressed the Cre reporter (data not shown), suggesting that *CD4cre* is deleting genes in a small fraction of cells other than T cells, which may impact the phenotype if the gene plays an essential role in other cell types.

## Discussion

The data presented here reveal two novel levels of chondrocyte regulation. First, ERK signaling in a cell that expresses CD4 is critical for regulating excess cartilage accumulation in the growth plate. Second, the presence of T cells delays the development of osteochondromas, demonstrating a critical impact of the immune system on cartilage homeostasis.

While there is a clear role for ERK signaling within chondrocytes to regulate proliferation and differentiation, it is likely that there are multiple levels of chondrocyte regulation. We propose that the growth of chondrocytes is regulated by signaling pathways within the chondrocytes, as well as external cues from cells within the environment. The fact that osteochondromas only developed in DKO^CD4^ mice, but not mice that only lack *Erk2* in CD4-expressing cells, suggests that the chondrocytes must also lack *Erk1* in order for osteochondromas to form. The absence of *Erk1* in the chondrocytes may render these cells more susceptible to ectopic accumulation; however, disease does not occur unless *Erk2* is also deleted in a cell type that expressed CD4 at one point during development.

Although chondrocytes and their progenitors are not known to express CD4, we observed *CD4cre* expression in a small fraction of chondrocytes in both WT^CD4^ and DKO^CD4^ mice, suggesting that a proportion of chondrocytes may be deficient in both *Erk1* and *Erk2*. However, our data show that the majority of the chondrocytes in DKO^CD4^ mice maintain *Erk2* expression. In fact, chondrocytes isolated from young mice showed no deletion of *Erk2* (Figure 4A). Furthermore, if both *Erk1* and *Erk2* were deleted in the majority of chondrocytes, we would expect to see elongated bones, deformed bones, or disorganized growth plates in DKO^CD4^ mice beginning at a very young age as others observed when ERK signaling was impaired specifically within the chondrocytes (7).

While it is clear that *Erk2* is not deleted in the majority of the chondrocytes in the DKO^CD4^ mice, osteochondromas can form when only a proportion of chondrocytes lack critical regulators such as *ptpn11* (SHP2) or *Ext1*. Mice with deletion of these genes in 10–50% of chondrocytes developed cartilaginous tumors composed of a mix of wild-type and mutated chondrocytes (18, 19). Although it is not confirmed in these models that deletion of these genes in a small fraction of chondrocytes caused osteochondromas, it is possible that deletion of *Erk2* in only a proportion of the chondrocytes in DKO^CD4^ mice induced osteochondromas. It is also conceivable that deletion of *Erk2* in cells other than chondrocytes regulates cartilage homeostasis. Sections of growth plates from the Cre reporter mice showed a significant population of cells that deleted the gene, but were not chondrocytes. These cells are in close proximity to the chondrocytes and could contribute to chondrocyte accumulation.

Whether the osteochondromas form due to deletion of *Erk2* in the chondrocytes or another subset of immune cells, our data show that the presence of T cells delays the onset and development of osteochondromas. This is surprising as it has not been shown that T cells, or any cell of the immune system, alter development of osteochondromas. T cells may impact osteochondroma function directly by suppressing tumor growth through cytolytic mechanisms. However, there is little evidence of infiltrating T cells in osteochondromas, and therefore, it is likely that the T cells affect the formation of osteochondromas indirectly by expressing a regulatory cytokine or influencing the development or function of a cell type that promotes osteochondroma formation.

Interestingly, deletion of Sos1/2 *via CD4cre* also caused development of osteochondromas similar to DKO^CD4^ mice (20). Sos-1/2 are Ras-guanine exchange factors that contribute to activation of ERK signaling (21). Deletion of Sos1/2 reduces ERK activation, and deletion of either of these genes *via CD4cre* results in spontaneous osteochondroma formation (20). These data highlight that this pathway is important in cells that expressed CD4 in order to regulate chondrocyte accumulation.

Together, these data suggest that although *Erk1* deletion may render chondrocytes more sensitive to ectopic accumulation, disease does not occur unless *Erk2* is also deleted in a cell type that expressed CD4 at one point during development. Importantly, the fact that T cells delay the onset and decrease the severity of osteochondromas reveals a previously unknown role for the immune system in regulating cartilage homeostasis.

## Ethics Statement

This study was carried out in accordance with the recommendations of IACUC. The protocol was approved by the St. Jude IACUC committee.

## Author Contributions

MW designed and performed research, analyzed data, and wrote the paper. AC and MC designed research, performed research, and analyzed data. CG, BE, and CC performed research and analyzed data. GP and JP contributed new reagents. PV analyzed data. MM designed and performed research, analyzed data, secured funding, and wrote the paper.

## Conflict of Interest Statement

The authors declare that the research was conducted in the absence of any commercial or financial relationships that could be construed as a potential conflict of interest.
