# Supplementary material for: Extracellular Signal-Regulated Kinase Signaling in CD4-Expressing Cells Inhibits Osteochondromas
Source: Front Immunol. 2017 May 1;8:482. doi: 10.3389/fimmu.2017.00482 (PMC5410564; doi:10.3389/fimmu.2017.00482)
Supplement: Supplementary file 1 [file image_1.pdf]

Supplementary Figure 1.

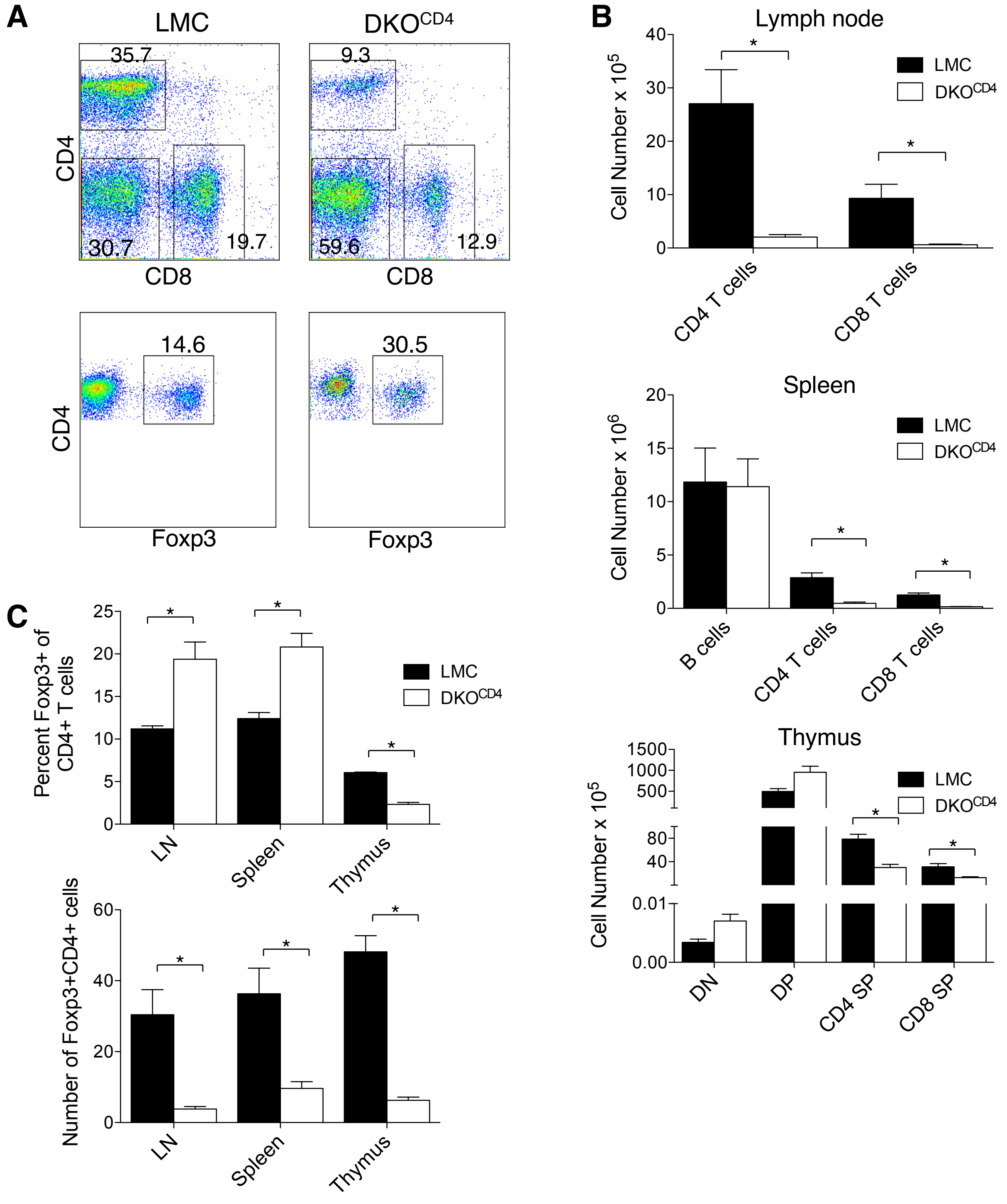

Figure S1. DKO^{CD4} mice have reduced numbers of T cells. Cells isolated from the lymph nodes, spleen, or thymus of DKO^{CD4} and LMC mice were stained with antibodies to CD4, CD8, CD19, CD44, CD62L, and Foxp3, then analyzed by flow cytometry. (A) Representative FACS plots from lymph nodes, with lower panels gated on CD4^{+} cells. (B) Quantification of immune cell subsets per organ in four week old LMC (n=3) and 129.DKO^{CD4} mice (n=5). These data are representative of two independent experiments. (C) The proportion and number of CD4^{+} FoxP3^{+} Treg cells in mice from *B*. Statistical analyses were performed using a Mann-Whitney test. *$P < 0.05$

Supplementary Figure 2.

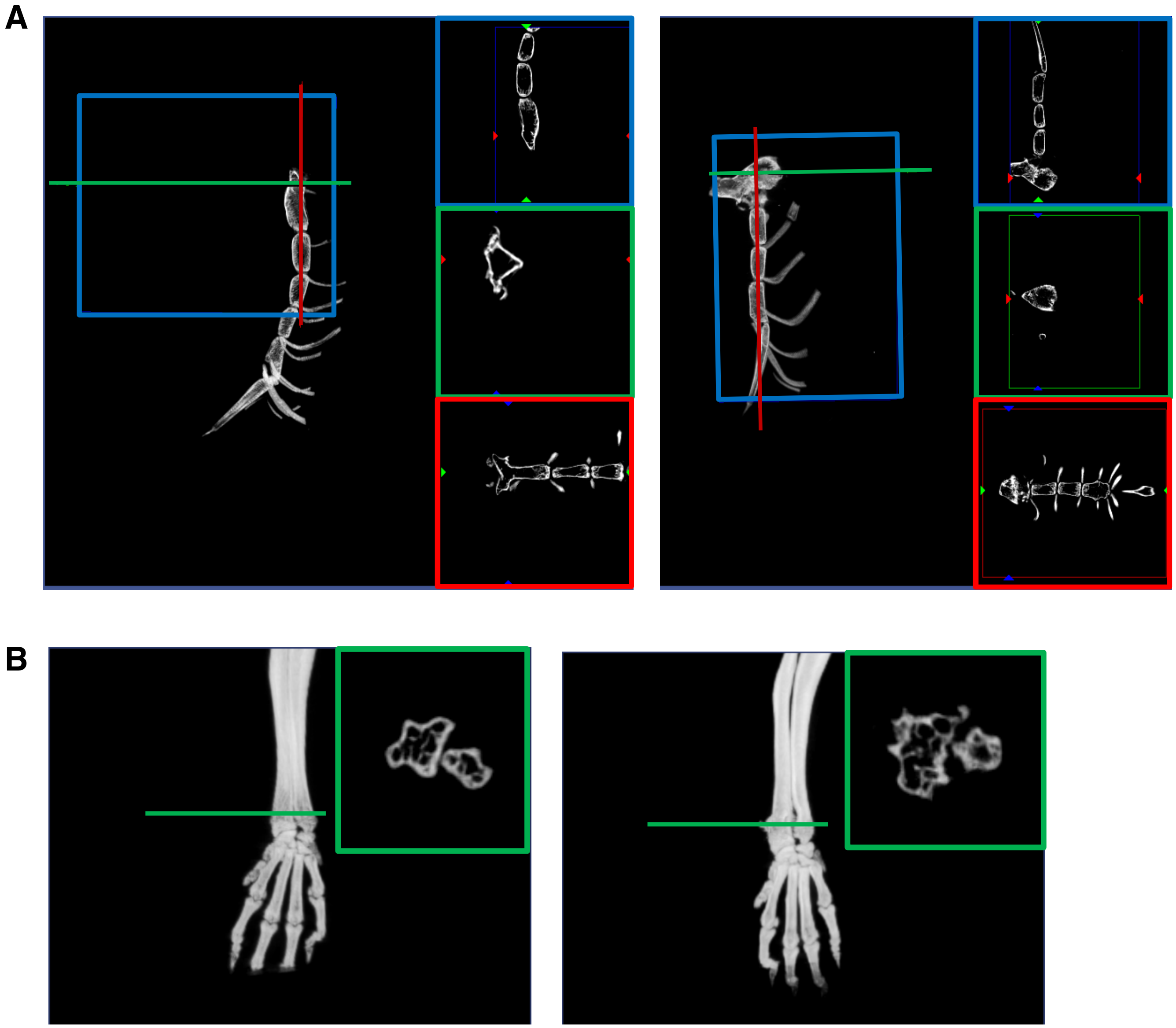

Figure S2. Lesions are continuous with the bone. *Ex vivo* CT MIP images of (**a**) sterna or (**b**) radii and ulnae showing the continuity of the marrow and cortices of the lesion with the bone. The location of the each slice is indicated by the colored lines, which correspond to the border surrounding the inset box.

Supplemental Figure 3

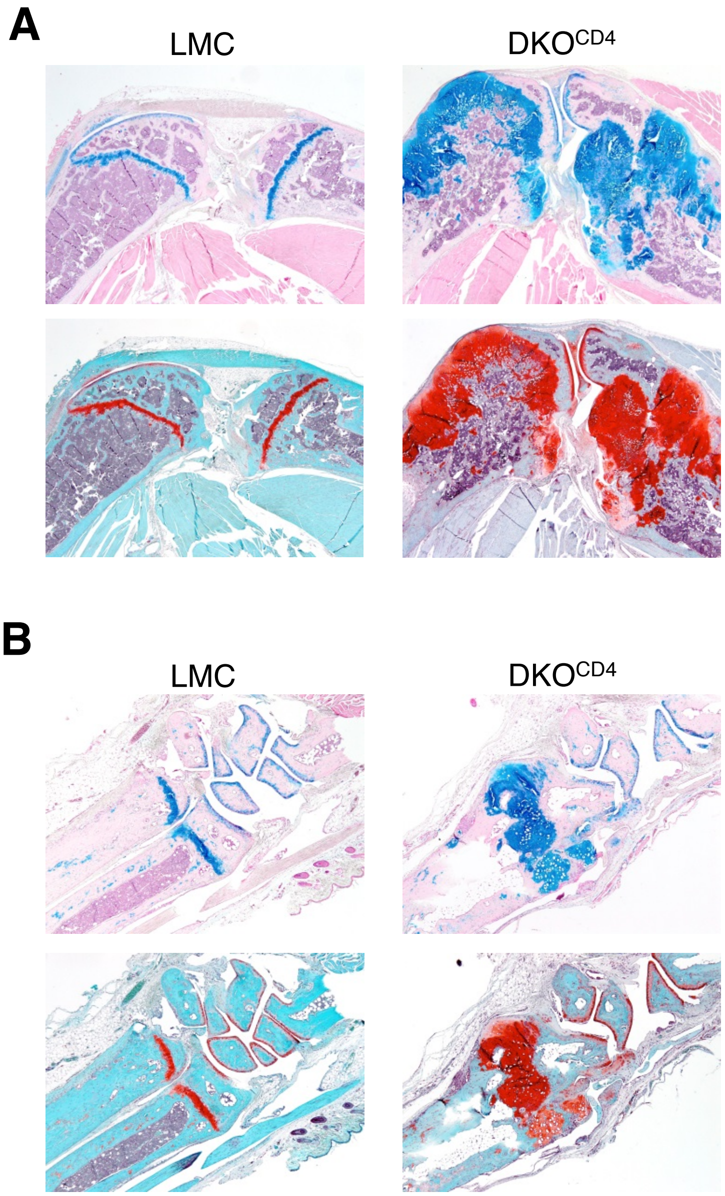

Figure S3. Chondrocytes accumulate in DKO^{CD4} mice and alter growth plate morphology. Sections of (A) tibiofemoral (2X) and (B) radiocarpal joints (4X) were stained with Alcian blue/hematoxylin (top) or Safarin O/Fast green (bottom) following decalcification.

Supplementary Figure 4

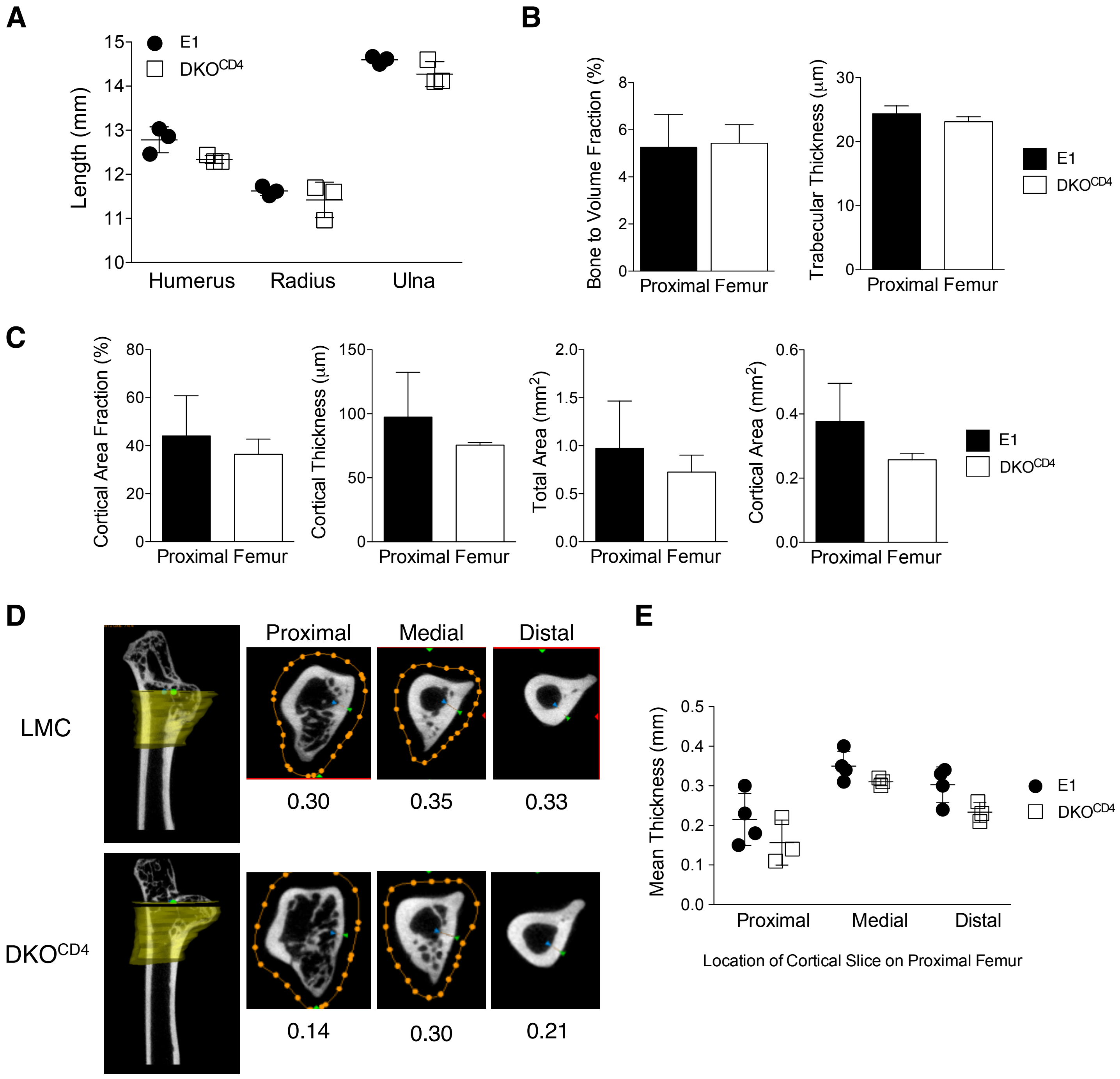

Figure S4. DKO^{CD4} mice do not display bone deformities or alteration in length. (A) Bone length was determined from *ex vivo* CT images of three E1 (*Erk1*$^{-/-}$) and three 129.DKO^{CD4} male mice. (B-C) *Ex vivo* CT analysis of the proximal femurs of four male E1 and three male 129.DKO^{CD4} mice at 28 weeks of age was used to determine (B) trabecular thickness and bone volume fractions as well as (C) cortical thickness and cortical area fractions. (D) *Ex vivo* CT MIP images of the proximal femurs of a representative E1 and DKO^{CD4} mouse with the three slices used to determine in-plane cortical bone thickness. Numbers below are indicative of the thickness, as measured by manual electronic calipers. (E) Quantification of the data from Figure S4D.

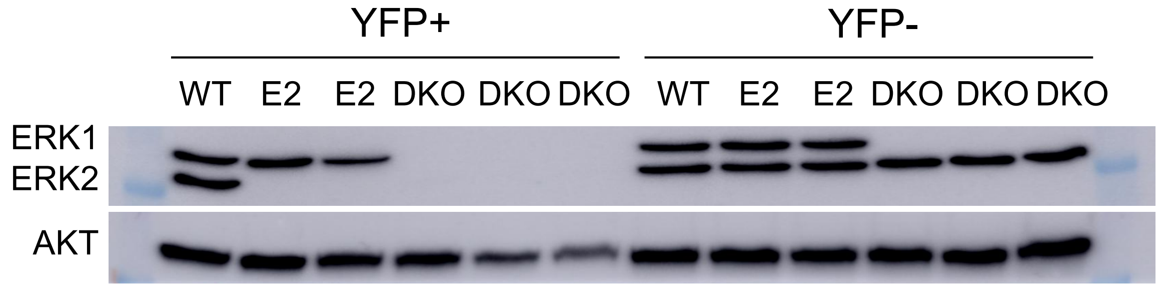

Figure S5. YFP expression reports *Erk2* deletion. YFP+ splenocytes were sorted from $Erk1^{+/+}.Erk2^{+/+}.YFP^{fl/fl}.CD4cre$+ (WT), $Erk1^{+/+}.Erk2^{fl/fl}.YFP^{fl/fl}.CD4cre$+ (E2), and $Erk1^{-/-}.Erk2^{fl/fl}.YFP^{fl/fl}.CD4cre$+ (DKO) mice. Cells were lysed and analyzed by western blot for ERK1 and ERK2 expression. AKT serves as a loading control.
